# 1-Benzyl-3,5-bis­(4-chloro­benzyl­idene)piperidin-4-one

**DOI:** 10.1107/S1600536811018587

**Published:** 2011-05-25

**Authors:** Volodymyr V. Nesterov, Sergey S. Sarkisov, Vladimir Shulaev, Vladimir N. Nesterov

**Affiliations:** a Department of Natural Sciences, New Mexico Highlands University, Las Vegas, NM 87701, USA; b SSS Optical Technologies, LLC, 515 Sparkman Drive, Suite 122, Huntsville, AL 35816, USA; c Department of Biological Sciences, University of North Texas, Denton, TX 76203, USA; d Department of Chemistry, University of North Texas, Denton, TX 76203, USA

## Abstract

The title compound, C_26_H_21_Cl_2_NO, crystallizes with two symmetry-independent mol­ecules (*A* and *B*) in the asymmetric unit. In both mol­ecules, the central heterocyclic ring adopts a sofa conformation. The dihedral angles between the planar part of this central heterocyclic ring [maximum deviations of 0.011 (1) and 0.036 (1) Å in mol­ecules *A* and *B*, respectively] and the two almost planar [maximum deviations of 0.020 (1) and 0.008 (1) Å in *A* and 0.007 (1) and 0.011 (1) in *B*] side-chain fragments that include the aromatic ring and bridging atoms are 20.1 (1) and 31.2 (1)° in mol­ecule *A*, and 26.4 (1) and 19.6 (1)° in mol­ecule *B*. The dihedral angles between the planar part of the heterocyclic ring and the benzyl substituent are 79.7 (1) and 53.2 (1)° in mol­ecules *A* and *B*, respectively. In the crystal, weak inter­molecular C—H⋯O hydrogen bonds link the two independent mol­ecules into dimers.

## Related literature

For non-linear optical organic compounds with two-photon absorption properties and potential biophotonic materials, see: Nesterov *et al.* (2003 ▶, 2007 ▶); Sarkisov *et al.* (2005 ▶). For the biological importance of 4-piperidone, see: Jia *et al.* (1988 ▶); Dimmock *et al.* (2001 ▶). For the synthesis of the title compound, see: Dimmock *et al.* (2001 ▶). For related structures, see: Nesterov *et al.* (2003 ▶, 2007 ▶, 2011 ▶). For details concerning weak hydrogen bonds, see: Desiraju & Steiner (1999 ▶). For van der Waals radii, see: Rowland & Taylor (1996 ▶).
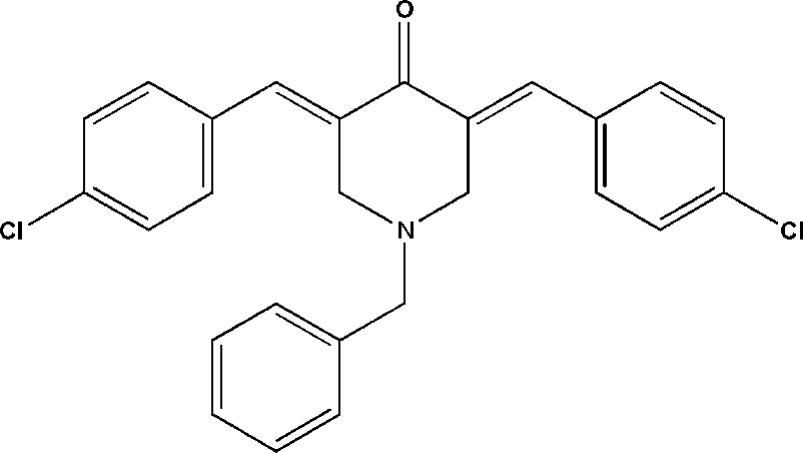


## Experimental

### 

#### Crystal data

C_26_H_21_Cl_2_NO
*M* *_r_* = 434.34
Triclinic, 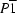

*a* = 12.504 (2) Å
*b* = 13.414 (4) Å
*c* = 14.763 (2) Å
α = 102.736 (3)°
β = 111.676 (2)°
γ = 104.066 (3)°
*V* = 2095.7 (8) Å^3^
*Z* = 4
Mo *K*α radiation
μ = 0.33 mm^−1^
*T* = 100 K
0.25 × 0.20 × 0.12 mm

#### Data collection

Bruker SMART APEXII CCD diffractometer
Absorption correction: multi-scan (*SADABS*; Bruker, 2001 ▶) *T*_min_ = 0.922, *T*_max_ = 0.962
24542 measured reflections
8251 independent reflections
7049 reflections with *I* > 2σ(*I*)
*R*_int_ = 0.024

#### Refinement

*R*[*F* ^2^ > 2σ(*F* ^2^)] = 0.039
*wR*(*F* ^2^) = 0.108
*S* = 1.02
8251 reflections
541 parameters
H-atom parameters constrained
Δρ_max_ = 0.79 e Å^−3^
Δρ_min_ = −0.48 e Å^−3^

### 

Data collection: *APEX2* (Bruker, 2007 ▶); cell refinement: *SAINT* (Bruker, 2007 ▶); data reduction: *SAINT*; program(s) used to solve structure: *SHELXS97* (Sheldrick, 2008 ▶); program(s) used to refine structure: *SHELXL97* (Sheldrick, 2008 ▶); molecular graphics: *SHELXTL* (Sheldrick, 2008 ▶); software used to prepare material for publication: *SHELXTL*.

## Supplementary Material

Crystal structure: contains datablocks I, global. DOI: 10.1107/S1600536811018587/fl2348sup1.cif

Structure factors: contains datablocks I. DOI: 10.1107/S1600536811018587/fl2348Isup2.hkl

Supplementary material file. DOI: 10.1107/S1600536811018587/fl2348Isup3.cml

Additional supplementary materials:  crystallographic information; 3D view; checkCIF report

## Figures and Tables

**Table 1:** Hydrogen-bond geometry (Å, °)

*D*—H⋯*A*	*D*—H	H⋯*A*	*D*⋯*A*	*D*—H⋯*A*
C16*B*—H16*B*⋯O1*A*	0.95	2.42	3.334 (2)	160
C14*A*—H14*A*⋯O1*B*	0.95	2.52	3.309 (2)	141
C16*A*—H16*A*⋯O1*B*	0.95	2.48	3.099 (2)	122

**Fig. 1.**
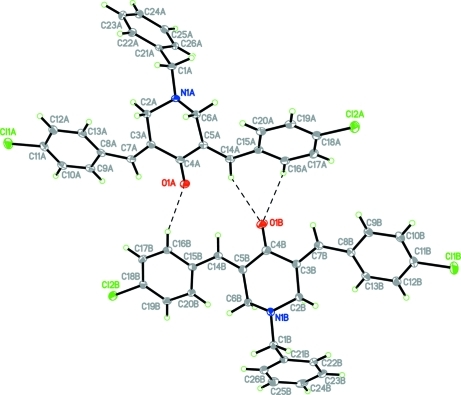
Perspective view of the asymmetric unit in (I), with hydrogen bonds shown as dashed lines. Displacement ellipsoids are shown at the 30% probability level.

**Fig. 2.**
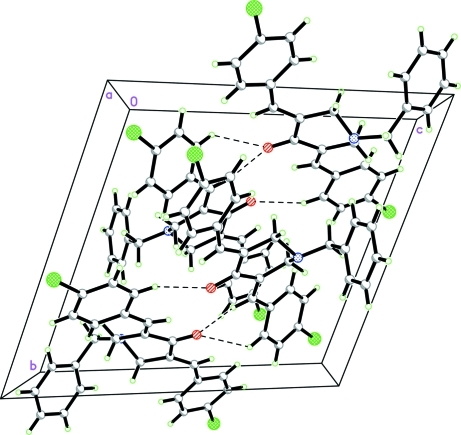
Projection of the crystal packing of the title compound along the a-axis. Dashed lines denote weak intermolecular C—H···O hydrogen bonds.

## supplementary crystallographic information

### Comment

Continuing our work on the synthesis and structural investigations of nonlinear optical organic compounds with two-photon absorption properties and potential biophotonic materials (Nesterov *et al.*, 2003; Nesterov *et al.* 2007; Nesterov *et al.*, 2011; Sarkisov *et al.*, 2005), we investigated the crystal structure of the title compound. This compound belongs to a group that has shown anticancer activity (Jia *et al.*, 1988; Dimmock *et al.*, 2001). It may also find application as an agent for locating cancer cells with two photon excited fluorescence and as a potential agent for a photodynamic treatment of cancer (Nesterov *et al.*, 2003; Sarkisov *et al.*, 2005).

The molecular structure of the title compound is illustrated in Fig. 1. The central heterocycle adopts a sofa conformation: atom N1A lies -0.721 (2) Å in (IA) and N1B lies 0.725 (2) Å in (IB) out of the central C_5_ plane [planar within 0.011 (1) and 0.036 (1) Å, respectively]. Dihedral angles between the flat part of the heterocycle (atoms C2A,C3A,C4A,C5A,C6A in (IA) and C2B,C3B,C4B,C5B,C6B in (IB) and the two almost planar fragments that include the Ph-ring and the bridging atoms are 20.1 (1) and 31.2 (1)° in (IA) for (C7A-C13A) and (C14A-C20A), respectively and 26.4 (1) and 19.6 (1)° for (C7B-C13B) and (C14B-C20B), respectively. Such nonplanarity might partly be caused by the presence of short intramolecular contacts H2AA···H13A and H6AB···H20A in (IA) and H2BB···H13B and H6BA···H20B in (IB) with distances 2.19 and 2.18 Å in (IA) and 2.14 and 2.22 Å in (IB), that are somewhat shorter than the doubled van der Waals radii of the H atom (Rowland & Taylor, 1996). Atoms N1A and N1B in the piperidone rings have a pyramidal coordination with the sum of bond angles equal to 331.4 (1) and 335.8 (1)°, while the methylene substituent connected to it occupies an equatorial position. The mutual orientations of the benzyl substituents and flat part of the heterocycles in both molecules are more different (dihedral angles are 79.7 (1) and 53.2 (1)°, respectively).

In the crystal there are several weak intermolecular C—H···O contacts (Table 1) that could be considered as weak hydrogen bonds (Desiraju & Steiner, 1999) that link (IA) and (IB) molecules into dimers (Fig. 2).

### Experimental

The title compound was obtained according to the literature procedure (Dimmock *et al.*, 2001) by the reaction of *p*-chlorobenzaldehyde with 1-benzyl-4-piperidone. The precipitate obtained was isolated and recrystallized from ethanol/acetonitrile [v/v = 50/50]; Mp. 424 K, yield 86%). The title compound was characterized by ^1^H and ^13^C NMR spectroscopy.

### Refinement

All C-bound H atoms were placed in idealized positions and allowed to ride on their parent atom: C—H = 0.95 and 0.99 Å for CH and CH_2_ H-atoms, respectively, with *U*_iso_(H) = k × *U*_eq_(C), where k = 1.2 for all H-atoms.

### Crystal data

**Table:**

C_26_H_21_Cl_2_NO	*Z* = 4
*M**_r_* = 434.34	*F*(000) = 904
Triclinic, *P*1	*D*_x_ = 1.377 Mg m^−^^3^
Hall symbol: -P 1	Mo *K*α radiation, λ = 0.71073 Å
*a* = 12.504 (2) Å	Cell parameters from 2357 reflections
*b* = 13.414 (4) Å	θ = 2.4–25.4°
*c* = 14.763 (2) Å	µ = 0.33 mm^−^^1^
α = 102.736 (3)°	*T* = 100 K
β = 111.676 (2)°	Plate, yellow
γ = 104.066 (3)°	0.25 × 0.20 × 0.12 mm
*V* = 2095.7 (8) Å^3^	

### Data collection

**Table:**

Bruker SMART APEXII CCD diffractometer	8251 independent reflections
Radiation source: fine-focus sealed tube	7049 reflections with *I* > 2σ(*I*)
graphite	*R*_int_ = 0.024
ω scans	θ_max_ = 26.0°, θ_min_ = 1.7°
Absorption correction: multi-scan (*SADABS*; Bruker, 2001)	*h* = −15→15
*T*_min_ = 0.922, *T*_max_ = 0.962	*k* = −16→16
24542 measured reflections	*l* = −18→18

### Refinement

**Table:**

Refinement on *F*^2^	Primary atom site location: structure-invariant direct methods
Least-squares matrix: full	Secondary atom site location: difference Fourier map
*R*[*F*^2^ > 2σ(*F*^2^)] = 0.039	Hydrogen site location: inferred from neighbouring sites
*wR*(*F*^2^) = 0.108	H-atom parameters constrained
*S* = 1.02	*w* = 1/[σ^2^(*F*_o_^2^) + (0.057*P*)^2^ + 1.110*P*] where *P* = (*F*_o_^2^ + 2*F*_c_^2^)/3
8251 reflections	(Δ/σ)_max_ = 0.001
541 parameters	Δρ_max_ = 0.79 e Å^−^^3^
0 restraints	Δρ_min_ = −0.48 e Å^−^^3^

### Special details

**Table:**

Geometry. All esds (except the esd in the dihedral angle between two l.s. planes) are estimated using the full covariance matrix. The cell esds are taken into account individually in the estimation of esds in distances, angles and torsion angles; correlations between esds in cell parameters are only used when they are defined by crystal symmetry. An approximate (isotropic) treatment of cell esds is used for estimating esds involving l.s. planes.
Refinement. Refinement of F^2^ against ALL reflections. The weighted R-factor wR and goodness of fit S are based on F^2^, conventional R-factors R are based on F, with F set to zero for negative F^2^. The threshold expression of F^2^ > 2sigma(F^2^) is used only for calculating R-factors(gt) etc. and is not relevant to the choice of reflections for refinement. R-factors based on F^2^ are statistically about twice as large as those based on F, and R- factors based on ALL data will be even larger.

### Fractional atomic coordinates and isotropic or equivalent isotropic displacement parameters (Å^2^)

**Table:**

	*x*	*y*	*z*	*U*_iso_*/*U*_eq_	
Cl1A	0.01606 (5)	0.78507 (4)	0.69670 (4)	0.03870 (13)	
Cl2A	0.65255 (5)	0.12893 (5)	0.08931 (4)	0.03854 (13)	
O1A	0.35806 (13)	0.34345 (11)	0.51320 (10)	0.0336 (3)	
N1A	0.24037 (13)	0.45157 (12)	0.27541 (11)	0.0214 (3)	
C1A	0.20424 (16)	0.49694 (15)	0.19088 (13)	0.0235 (4)	
H1AA	0.2213	0.4597	0.1348	0.028*	
H1AB	0.2548	0.5758	0.2178	0.028*	
C2A	0.24894 (16)	0.52293 (14)	0.37107 (13)	0.0225 (4)	
H2AA	0.1714	0.5376	0.3558	0.027*	
H2AB	0.3172	0.5937	0.3974	0.027*	
C3A	0.27142 (16)	0.46956 (14)	0.45281 (13)	0.0225 (4)	
C4A	0.33892 (16)	0.39154 (14)	0.44991 (13)	0.0237 (4)	
C5A	0.38468 (15)	0.37528 (14)	0.36967 (13)	0.0226 (4)	
C6A	0.35812 (16)	0.43693 (15)	0.29532 (13)	0.0230 (4)	
H6AA	0.4249	0.5093	0.3251	0.028*	
H6AB	0.3550	0.3959	0.2293	0.028*	
C7A	0.23584 (16)	0.48474 (15)	0.52962 (13)	0.0243 (4)	
H7AA	0.2517	0.4389	0.5698	0.029*	
C8A	0.17781 (16)	0.55822 (15)	0.56197 (13)	0.0242 (4)	
C9A	0.12682 (16)	0.53599 (16)	0.62905 (13)	0.0273 (4)	
H9AA	0.1272	0.4722	0.6470	0.033*	
C10A	0.07631 (16)	0.60382 (16)	0.66965 (14)	0.0279 (4)	
H10A	0.0426	0.5874	0.7150	0.033*	
C11A	0.07585 (17)	0.69624 (16)	0.64286 (14)	0.0283 (4)	
C12A	0.12458 (17)	0.72161 (15)	0.57642 (14)	0.0287 (4)	
H12A	0.1224	0.7849	0.5581	0.034*	
C13A	0.17646 (17)	0.65334 (15)	0.53720 (13)	0.0265 (4)	
H13A	0.2115	0.6712	0.4931	0.032*	
C14A	0.44035 (15)	0.30143 (15)	0.36640 (13)	0.0238 (4)	
H14A	0.4473	0.2674	0.4173	0.029*	
C15A	0.49216 (15)	0.26528 (15)	0.29595 (13)	0.0235 (4)	
C16A	0.49531 (16)	0.15947 (15)	0.27926 (14)	0.0256 (4)	
H16A	0.4647	0.1160	0.3133	0.031*	
C17A	0.54175 (17)	0.11651 (16)	0.21450 (15)	0.0285 (4)	
H17A	0.5406	0.0436	0.2023	0.034*	
C18A	0.59003 (16)	0.18160 (16)	0.16772 (14)	0.0271 (4)	
C19A	0.59069 (16)	0.28780 (15)	0.18407 (14)	0.0269 (4)	
H19A	0.6250	0.3321	0.1524	0.032*	
C20A	0.54096 (16)	0.32860 (15)	0.24702 (13)	0.0250 (4)	
H20A	0.5399	0.4007	0.2572	0.030*	
C21A	0.06923 (16)	0.48300 (14)	0.14668 (12)	0.0212 (3)	
C22A	0.03196 (17)	0.57277 (14)	0.14847 (14)	0.0265 (4)	
H22A	0.0921	0.6446	0.1806	0.032*	
C23A	−0.09251 (18)	0.55824 (15)	0.10361 (14)	0.0300 (4)	
H23A	−0.1169	0.6201	0.1046	0.036*	
C24A	−0.18115 (17)	0.45380 (15)	0.05750 (14)	0.0275 (4)	
H24A	−0.2662	0.4438	0.0266	0.033*	
C25A	−0.14470 (17)	0.36398 (15)	0.05688 (13)	0.0257 (4)	
H25A	−0.2051	0.2923	0.0263	0.031*	
C26A	−0.02083 (17)	0.37834 (14)	0.10062 (13)	0.0240 (4)	
H26A	0.0032	0.3163	0.0993	0.029*	
Cl1B	0.95734 (5)	−0.23822 (4)	0.32282 (4)	0.03777 (13)	
Cl2B	0.37099 (5)	0.37742 (4)	0.99889 (4)	0.03390 (13)	
O1B	0.56754 (19)	0.16150 (17)	0.50449 (12)	0.0619 (6)	
N1B	0.80808 (13)	0.13829 (11)	0.75624 (11)	0.0214 (3)	
C1B	0.90276 (16)	0.13447 (14)	0.85070 (13)	0.0231 (4)	
H1BA	0.9251	0.2006	0.9096	0.028*	
H1BB	0.9775	0.1383	0.8406	0.028*	
C2B	0.77495 (16)	0.04704 (14)	0.66319 (13)	0.0218 (3)	
H2BA	0.7250	−0.0217	0.6647	0.026*	
H2BB	0.8506	0.0382	0.6615	0.026*	
C3B	0.70258 (17)	0.06941 (14)	0.56795 (13)	0.0244 (4)	
C4B	0.62426 (19)	0.13581 (17)	0.57702 (15)	0.0321 (4)	
C5B	0.61373 (16)	0.16622 (14)	0.67621 (13)	0.0236 (4)	
C6B	0.69847 (16)	0.14481 (14)	0.76757 (13)	0.0221 (4)	
H6BA	0.7233	0.2045	0.8326	0.026*	
H6BB	0.6544	0.0753	0.7718	0.026*	
C7B	0.70371 (17)	0.03605 (15)	0.47530 (14)	0.0259 (4)	
H7BA	0.6560	0.0600	0.4238	0.031*	
C8B	0.76759 (16)	−0.03161 (14)	0.44228 (13)	0.0242 (4)	
C9B	0.79157 (17)	−0.02325 (15)	0.35788 (14)	0.0260 (4)	
H9BA	0.7661	0.0258	0.3247	0.031*	
C10B	0.85128 (18)	−0.08460 (15)	0.32190 (14)	0.0288 (4)	
H10B	0.8684	−0.0767	0.2658	0.035*	
C11B	0.88558 (17)	−0.15780 (15)	0.36921 (14)	0.0274 (4)	
C12B	0.86061 (17)	−0.17053 (15)	0.45097 (14)	0.0273 (4)	
H12B	0.8835	−0.2218	0.4820	0.033*	
C13B	0.80220 (17)	−0.10792 (14)	0.48681 (13)	0.0254 (4)	
H13B	0.7851	−0.1167	0.5427	0.030*	
C14B	0.52956 (16)	0.21299 (14)	0.67633 (13)	0.0241 (4)	
H14B	0.4869	0.2238	0.6133	0.029*	
C15B	0.49337 (15)	0.24968 (14)	0.75771 (13)	0.0216 (3)	
C16B	0.43215 (16)	0.32462 (14)	0.74851 (14)	0.0235 (4)	
H16B	0.4161	0.3487	0.6906	0.028*	
C17B	0.39458 (16)	0.36416 (15)	0.82178 (14)	0.0256 (4)	
H17B	0.3554	0.4164	0.8154	0.031*	
C18B	0.41504 (16)	0.32627 (15)	0.90467 (13)	0.0235 (4)	
C19B	0.47058 (16)	0.24907 (15)	0.91413 (13)	0.0245 (4)	
H19B	0.4811	0.2217	0.9697	0.029*	
C20B	0.51062 (16)	0.21212 (14)	0.84192 (13)	0.0234 (4)	
H20B	0.5505	0.1604	0.8494	0.028*	
C21B	0.86682 (15)	0.03449 (14)	0.88084 (13)	0.0223 (4)	
C22B	0.88113 (16)	−0.06166 (15)	0.83723 (14)	0.0265 (4)	
H22B	0.9155	−0.0639	0.7895	0.032*	
C23B	0.84610 (18)	−0.15432 (16)	0.86226 (15)	0.0324 (4)	
H23B	0.8552	−0.2196	0.8310	0.039*	
C24B	0.79766 (18)	−0.15083 (17)	0.93324 (16)	0.0360 (5)	
H24B	0.7741	−0.2137	0.9511	0.043*	
C25B	0.78367 (18)	−0.05629 (18)	0.97777 (16)	0.0344 (4)	
H25B	0.7504	−0.0542	1.0262	0.041*	
C26B	0.81808 (17)	0.03602 (16)	0.95213 (14)	0.0278 (4)	
H26B	0.8083	0.1009	0.9835	0.033*	

### Atomic displacement parameters (Å^2^)

**Table:**

	*U*^11^	*U*^22^	*U*^33^	*U*^12^	*U*^13^	*U*^23^
Cl1A	0.0431 (3)	0.0438 (3)	0.0363 (3)	0.0205 (2)	0.0243 (2)	0.0098 (2)
Cl2A	0.0449 (3)	0.0511 (3)	0.0420 (3)	0.0289 (2)	0.0311 (2)	0.0227 (2)
O1A	0.0478 (8)	0.0417 (8)	0.0285 (7)	0.0268 (7)	0.0218 (6)	0.0227 (6)
N1A	0.0245 (7)	0.0260 (7)	0.0169 (7)	0.0114 (6)	0.0089 (6)	0.0108 (6)
C1A	0.0262 (9)	0.0290 (9)	0.0191 (8)	0.0118 (7)	0.0101 (7)	0.0129 (7)
C2A	0.0246 (9)	0.0240 (9)	0.0195 (8)	0.0090 (7)	0.0095 (7)	0.0084 (7)
C3A	0.0226 (8)	0.0219 (8)	0.0186 (8)	0.0054 (7)	0.0065 (7)	0.0066 (7)
C4A	0.0256 (9)	0.0253 (9)	0.0179 (8)	0.0078 (7)	0.0075 (7)	0.0085 (7)
C5A	0.0211 (8)	0.0254 (9)	0.0200 (8)	0.0080 (7)	0.0071 (7)	0.0095 (7)
C6A	0.0223 (8)	0.0289 (9)	0.0208 (8)	0.0099 (7)	0.0100 (7)	0.0124 (7)
C7A	0.0236 (9)	0.0270 (9)	0.0193 (8)	0.0068 (7)	0.0072 (7)	0.0095 (7)
C8A	0.0219 (8)	0.0273 (9)	0.0158 (8)	0.0050 (7)	0.0053 (7)	0.0031 (7)
C9A	0.0249 (9)	0.0310 (10)	0.0195 (8)	0.0045 (8)	0.0065 (7)	0.0088 (7)
C10A	0.0244 (9)	0.0356 (10)	0.0201 (9)	0.0066 (8)	0.0101 (7)	0.0075 (8)
C11A	0.0246 (9)	0.0324 (10)	0.0224 (9)	0.0081 (8)	0.0100 (7)	0.0023 (8)
C12A	0.0305 (10)	0.0265 (9)	0.0254 (9)	0.0076 (8)	0.0114 (8)	0.0070 (7)
C13A	0.0298 (9)	0.0279 (9)	0.0198 (8)	0.0064 (8)	0.0119 (7)	0.0073 (7)
C14A	0.0211 (8)	0.0292 (9)	0.0213 (8)	0.0077 (7)	0.0086 (7)	0.0122 (7)
C15A	0.0183 (8)	0.0300 (9)	0.0204 (8)	0.0097 (7)	0.0051 (7)	0.0105 (7)
C16A	0.0241 (9)	0.0319 (10)	0.0244 (9)	0.0119 (8)	0.0111 (7)	0.0135 (8)
C17A	0.0298 (10)	0.0304 (10)	0.0312 (10)	0.0148 (8)	0.0152 (8)	0.0139 (8)
C18A	0.0219 (9)	0.0399 (11)	0.0249 (9)	0.0153 (8)	0.0122 (7)	0.0134 (8)
C19A	0.0208 (9)	0.0337 (10)	0.0263 (9)	0.0081 (7)	0.0086 (7)	0.0159 (8)
C20A	0.0209 (8)	0.0280 (9)	0.0227 (9)	0.0081 (7)	0.0060 (7)	0.0099 (7)
C21A	0.0271 (9)	0.0259 (9)	0.0132 (7)	0.0113 (7)	0.0091 (7)	0.0090 (7)
C22A	0.0307 (10)	0.0207 (9)	0.0252 (9)	0.0081 (7)	0.0094 (8)	0.0091 (7)
C23A	0.0363 (10)	0.0264 (9)	0.0298 (10)	0.0183 (8)	0.0128 (8)	0.0097 (8)
C24A	0.0260 (9)	0.0333 (10)	0.0245 (9)	0.0141 (8)	0.0106 (7)	0.0098 (8)
C25A	0.0289 (9)	0.0238 (9)	0.0211 (9)	0.0072 (7)	0.0098 (7)	0.0066 (7)
C26A	0.0333 (10)	0.0214 (8)	0.0192 (8)	0.0134 (7)	0.0111 (7)	0.0075 (7)
Cl1B	0.0470 (3)	0.0375 (3)	0.0438 (3)	0.0213 (2)	0.0317 (2)	0.0141 (2)
Cl2B	0.0409 (3)	0.0470 (3)	0.0327 (3)	0.0277 (2)	0.0248 (2)	0.0200 (2)
O1B	0.0977 (14)	0.1087 (15)	0.0391 (9)	0.0861 (13)	0.0465 (10)	0.0507 (10)
N1B	0.0246 (7)	0.0225 (7)	0.0189 (7)	0.0097 (6)	0.0109 (6)	0.0070 (6)
C1B	0.0229 (8)	0.0234 (9)	0.0210 (8)	0.0080 (7)	0.0089 (7)	0.0060 (7)
C2B	0.0238 (8)	0.0214 (8)	0.0206 (8)	0.0084 (7)	0.0106 (7)	0.0065 (7)
C3B	0.0293 (9)	0.0253 (9)	0.0224 (9)	0.0111 (7)	0.0129 (7)	0.0111 (7)
C4B	0.0419 (11)	0.0417 (11)	0.0281 (10)	0.0248 (9)	0.0209 (9)	0.0197 (9)
C5B	0.0280 (9)	0.0244 (9)	0.0221 (9)	0.0104 (7)	0.0128 (7)	0.0105 (7)
C6B	0.0265 (9)	0.0239 (9)	0.0202 (8)	0.0114 (7)	0.0126 (7)	0.0091 (7)
C7B	0.0285 (9)	0.0281 (9)	0.0231 (9)	0.0107 (8)	0.0119 (8)	0.0109 (7)
C8B	0.0250 (9)	0.0241 (9)	0.0195 (8)	0.0061 (7)	0.0095 (7)	0.0046 (7)
C9B	0.0311 (10)	0.0251 (9)	0.0222 (9)	0.0086 (8)	0.0131 (8)	0.0086 (7)
C10B	0.0338 (10)	0.0287 (9)	0.0246 (9)	0.0062 (8)	0.0178 (8)	0.0075 (8)
C11B	0.0272 (9)	0.0255 (9)	0.0284 (9)	0.0074 (7)	0.0155 (8)	0.0040 (7)
C12B	0.0306 (10)	0.0246 (9)	0.0245 (9)	0.0086 (8)	0.0119 (8)	0.0066 (7)
C13B	0.0297 (9)	0.0259 (9)	0.0197 (8)	0.0076 (7)	0.0125 (7)	0.0065 (7)
C14B	0.0276 (9)	0.0263 (9)	0.0212 (8)	0.0108 (7)	0.0107 (7)	0.0120 (7)
C15B	0.0186 (8)	0.0230 (8)	0.0210 (8)	0.0056 (7)	0.0073 (7)	0.0084 (7)
C16B	0.0227 (8)	0.0288 (9)	0.0240 (9)	0.0103 (7)	0.0113 (7)	0.0154 (7)
C17B	0.0237 (9)	0.0288 (9)	0.0303 (9)	0.0145 (7)	0.0126 (8)	0.0143 (8)
C18B	0.0223 (8)	0.0284 (9)	0.0230 (9)	0.0101 (7)	0.0126 (7)	0.0091 (7)
C19B	0.0271 (9)	0.0292 (9)	0.0214 (9)	0.0121 (8)	0.0113 (7)	0.0130 (7)
C20B	0.0262 (9)	0.0245 (9)	0.0229 (9)	0.0122 (7)	0.0110 (7)	0.0110 (7)
C21B	0.0183 (8)	0.0251 (9)	0.0200 (8)	0.0084 (7)	0.0049 (7)	0.0071 (7)
C22B	0.0255 (9)	0.0302 (10)	0.0243 (9)	0.0142 (8)	0.0091 (7)	0.0095 (8)
C23B	0.0295 (10)	0.0264 (10)	0.0329 (10)	0.0136 (8)	0.0035 (8)	0.0093 (8)
C24B	0.0273 (10)	0.0338 (11)	0.0412 (12)	0.0067 (8)	0.0061 (9)	0.0235 (9)
C25B	0.0297 (10)	0.0465 (12)	0.0328 (10)	0.0136 (9)	0.0154 (9)	0.0212 (9)
C26B	0.0283 (9)	0.0334 (10)	0.0253 (9)	0.0148 (8)	0.0122 (8)	0.0119 (8)

### Geometric parameters (Å, °)

**Table:**

Cl1A—C11A	1.7507 (19)	Cl1B—C11B	1.7427 (19)
Cl2A—C18A	1.7398 (18)	Cl2B—C18B	1.7430 (18)
O1A—C4A	1.234 (2)	O1B—C4B	1.220 (2)
N1A—C6A	1.462 (2)	N1B—C6B	1.461 (2)
N1A—C2A	1.471 (2)	N1B—C2B	1.466 (2)
N1A—C1A	1.478 (2)	N1B—C1B	1.480 (2)
C1A—C21A	1.513 (2)	C1B—C21B	1.522 (2)
C1A—H1AA	0.9900	C1B—H1BA	0.9900
C1A—H1AB	0.9900	C1B—H1BB	0.9900
C2A—C3A	1.509 (2)	C2B—C3B	1.505 (2)
C2A—H2AA	0.9900	C2B—H2BA	0.9900
C2A—H2AB	0.9900	C2B—H2BB	0.9900
C3A—C7A	1.357 (2)	C3B—C7B	1.349 (2)
C3A—C4A	1.499 (2)	C3B—C4B	1.496 (3)
C4A—O1A	1.234 (2)	C4B—O1B	1.220 (2)
C4A—C5A	1.492 (2)	C4B—C5B	1.494 (2)
C5A—C14A	1.344 (3)	C5B—C14B	1.350 (2)
C5A—C6A	1.505 (2)	C5B—C6B	1.512 (2)
C6A—H6AA	0.9900	C6B—H6BA	0.9900
C6A—H6AB	0.9900	C6B—H6BB	0.9900
C7A—C8A	1.457 (3)	C7B—C8B	1.464 (3)
C7A—H7AA	0.9500	C7B—H7BA	0.9500
C8A—C13A	1.405 (3)	C8B—C13B	1.403 (3)
C8A—C9A	1.407 (3)	C8B—C9B	1.406 (2)
C9A—C10A	1.380 (3)	C9B—C10B	1.384 (3)
C9A—H9AA	0.9500	C9B—H9BA	0.9500
C10A—C11A	1.382 (3)	C10B—C11B	1.386 (3)
C10A—H10A	0.9500	C10B—H10B	0.9500
C11A—C12A	1.392 (3)	C11B—C12B	1.388 (3)
C12A—C13A	1.388 (3)	C12B—C13B	1.381 (3)
C12A—H12A	0.9500	C12B—H12B	0.9500
C13A—H13A	0.9500	C13B—H13B	0.9500
C14A—C15A	1.471 (2)	C14B—C15B	1.463 (2)
C14A—H14A	0.9500	C14B—H14B	0.9500
C15A—C16A	1.399 (3)	C15B—C16B	1.404 (2)
C15A—C20A	1.400 (3)	C15B—C20B	1.406 (2)
C16A—C17A	1.383 (3)	C16B—C17B	1.384 (2)
C16A—H16A	0.9500	C16B—H16B	0.9500
C17A—C18A	1.386 (3)	C17B—C18B	1.388 (2)
C17A—H17A	0.9500	C17B—H17B	0.9500
C18A—C19A	1.389 (3)	C18B—C19B	1.384 (3)
C19A—C20A	1.385 (3)	C19B—C20B	1.384 (2)
C19A—H19A	0.9500	C19B—H19B	0.9500
C20A—H20A	0.9500	C20B—H20B	0.9500
C21A—C22A	1.391 (2)	C21B—C22B	1.393 (3)
C21A—C26A	1.395 (2)	C21B—C26B	1.396 (2)
C22A—C23A	1.390 (3)	C22B—C23B	1.390 (3)
C22A—H22A	0.9500	C22B—H22B	0.9500
C23A—C24A	1.386 (3)	C23B—C24B	1.389 (3)
C23A—H23A	0.9500	C23B—H23B	0.9500
C24A—C25A	1.387 (3)	C24B—C25B	1.377 (3)
C24A—H24A	0.9500	C24B—H24B	0.9500
C25A—C26A	1.384 (3)	C25B—C26B	1.389 (3)
C25A—H25A	0.9500	C25B—H25B	0.9500
C26A—H26A	0.9500	C26B—H26B	0.9500
			
C6A—N1A—C2A	109.92 (13)	C6B—N1B—C2B	110.96 (13)
C6A—N1A—C1A	110.49 (13)	C6B—N1B—C1B	112.52 (13)
C2A—N1A—C1A	111.03 (13)	C2B—N1B—C1B	112.25 (13)
N1A—C1A—C21A	112.10 (14)	N1B—C1B—C21B	116.12 (14)
N1A—C1A—H1AA	109.2	N1B—C1B—H1BA	108.3
C21A—C1A—H1AA	109.2	C21B—C1B—H1BA	108.3
N1A—C1A—H1AB	109.2	N1B—C1B—H1BB	108.3
C21A—C1A—H1AB	109.2	C21B—C1B—H1BB	108.3
H1AA—C1A—H1AB	107.9	H1BA—C1B—H1BB	107.4
N1A—C2A—C3A	110.47 (14)	N1B—C2B—C3B	109.53 (14)
N1A—C2A—H2AA	109.6	N1B—C2B—H2BA	109.8
C3A—C2A—H2AA	109.6	C3B—C2B—H2BA	109.8
N1A—C2A—H2AB	109.6	N1B—C2B—H2BB	109.8
C3A—C2A—H2AB	109.6	C3B—C2B—H2BB	109.8
H2AA—C2A—H2AB	108.1	H2BA—C2B—H2BB	108.2
C7A—C3A—C4A	115.94 (16)	C7B—C3B—C4B	117.08 (16)
C7A—C3A—C2A	126.30 (16)	C7B—C3B—C2B	125.51 (16)
C4A—C3A—C2A	117.76 (15)	C4B—C3B—C2B	117.40 (15)
O1A—C4A—C5A	120.82 (16)	O1B—C4B—C5B	120.89 (18)
O1A—C4A—C5A	120.82 (16)	O1B—C4B—C5B	120.89 (18)
O1A—C4A—C3A	120.99 (16)	O1B—C4B—C3B	121.05 (17)
O1A—C4A—C3A	120.99 (16)	O1B—C4B—C3B	121.05 (17)
C5A—C4A—C3A	118.17 (15)	C5B—C4B—C3B	118.02 (16)
C14A—C5A—C4A	116.67 (16)	C14B—C5B—C4B	116.11 (16)
C14A—C5A—C6A	125.41 (16)	C14B—C5B—C6B	125.67 (16)
C4A—C5A—C6A	117.80 (15)	C4B—C5B—C6B	118.20 (15)
N1A—C6A—C5A	110.04 (14)	N1B—C6B—C5B	110.34 (14)
N1A—C6A—H6AA	109.7	N1B—C6B—H6BA	109.6
C5A—C6A—H6AA	109.7	C5B—C6B—H6BA	109.6
N1A—C6A—H6AB	109.7	N1B—C6B—H6BB	109.6
C5A—C6A—H6AB	109.7	C5B—C6B—H6BB	109.6
H6AA—C6A—H6AB	108.2	H6BA—C6B—H6BB	108.1
C3A—C7A—C8A	131.99 (17)	C3B—C7B—C8B	129.59 (17)
C3A—C7A—H7AA	114.0	C3B—C7B—H7BA	115.2
C8A—C7A—H7AA	114.0	C8B—C7B—H7BA	115.2
C13A—C8A—C9A	117.67 (17)	C13B—C8B—C9B	117.56 (16)
C13A—C8A—C7A	125.01 (16)	C13B—C8B—C7B	124.16 (16)
C9A—C8A—C7A	117.16 (17)	C9B—C8B—C7B	118.23 (16)
C10A—C9A—C8A	122.13 (18)	C10B—C9B—C8B	121.70 (17)
C10A—C9A—H9AA	118.9	C10B—C9B—H9BA	119.1
C8A—C9A—H9AA	118.9	C8B—C9B—H9BA	119.1
C9A—C10A—C11A	118.51 (17)	C9B—C10B—C11B	118.80 (17)
C9A—C10A—H10A	120.7	C9B—C10B—H10B	120.6
C11A—C10A—H10A	120.7	C11B—C10B—H10B	120.6
C10A—C11A—C12A	121.59 (17)	C10B—C11B—C12B	121.21 (17)
C10A—C11A—Cl1A	118.99 (14)	C10B—C11B—Cl1B	119.43 (14)
C12A—C11A—Cl1A	119.40 (15)	C12B—C11B—Cl1B	119.33 (15)
C13A—C12A—C11A	119.25 (18)	C13B—C12B—C11B	119.37 (17)
C13A—C12A—H12A	120.4	C13B—C12B—H12B	120.3
C11A—C12A—H12A	120.4	C11B—C12B—H12B	120.3
C12A—C13A—C8A	120.83 (17)	C12B—C13B—C8B	121.32 (17)
C12A—C13A—H13A	119.6	C12B—C13B—H13B	119.3
C8A—C13A—H13A	119.6	C8B—C13B—H13B	119.3
C5A—C14A—C15A	129.81 (16)	C5B—C14B—C15B	130.51 (16)
C5A—C14A—H14A	115.1	C5B—C14B—H14B	114.7
C15A—C14A—H14A	115.1	C15B—C14B—H14B	114.7
C16A—C15A—C20A	117.86 (17)	C16B—C15B—C20B	117.61 (16)
C16A—C15A—C14A	116.56 (16)	C16B—C15B—C14B	117.40 (15)
C20A—C15A—C14A	125.56 (17)	C20B—C15B—C14B	124.93 (16)
C17A—C16A—C15A	121.75 (17)	C17B—C16B—C15B	121.57 (16)
C17A—C16A—H16A	119.1	C17B—C16B—H16B	119.2
C15A—C16A—H16A	119.1	C15B—C16B—H16B	119.2
C16A—C17A—C18A	118.90 (18)	C16B—C17B—C18B	118.99 (16)
C16A—C17A—H17A	120.5	C16B—C17B—H17B	120.5
C18A—C17A—H17A	120.5	C18B—C17B—H17B	120.5
C17A—C18A—C19A	120.95 (17)	C19B—C18B—C17B	121.11 (16)
C17A—C18A—Cl2A	118.89 (15)	C19B—C18B—Cl2B	119.42 (14)
C19A—C18A—Cl2A	120.16 (14)	C17B—C18B—Cl2B	119.47 (14)
C20A—C19A—C18A	119.41 (16)	C20B—C19B—C18B	119.43 (16)
C20A—C19A—H19A	120.3	C20B—C19B—H19B	120.3
C18A—C19A—H19A	120.3	C18B—C19B—H19B	120.3
C19A—C20A—C15A	121.08 (17)	C19B—C20B—C15B	121.20 (16)
C19A—C20A—H20A	119.5	C19B—C20B—H20B	119.4
C15A—C20A—H20A	119.5	C15B—C20B—H20B	119.4
C22A—C21A—C26A	118.64 (16)	C22B—C21B—C26B	118.14 (17)
C22A—C21A—C1A	121.36 (16)	C22B—C21B—C1B	120.62 (16)
C26A—C21A—C1A	119.98 (15)	C26B—C21B—C1B	121.24 (16)
C23A—C22A—C21A	120.60 (17)	C23B—C22B—C21B	121.22 (18)
C23A—C22A—H22A	119.7	C23B—C22B—H22B	119.4
C21A—C22A—H22A	119.7	C21B—C22B—H22B	119.4
C24A—C23A—C22A	120.22 (17)	C24B—C23B—C22B	119.57 (18)
C24A—C23A—H23A	119.9	C24B—C23B—H23B	120.2
C22A—C23A—H23A	119.9	C22B—C23B—H23B	120.2
C23A—C24A—C25A	119.49 (17)	C25B—C24B—C23B	120.06 (18)
C23A—C24A—H24A	120.3	C25B—C24B—H24B	120.0
C25A—C24A—H24A	120.3	C23B—C24B—H24B	120.0
C26A—C25A—C24A	120.30 (17)	C24B—C25B—C26B	120.25 (19)
C26A—C25A—H25A	119.9	C24B—C25B—H25B	119.9
C24A—C25A—H25A	119.8	C26B—C25B—H25B	119.9
C25A—C26A—C21A	120.73 (16)	C25B—C26B—C21B	120.76 (18)
C25A—C26A—H26A	119.6	C25B—C26B—H26B	119.6
C21A—C26A—H26A	119.6	C21B—C26B—H26B	119.6
			
C6A—N1A—C1A—C21A	163.42 (14)	C6B—N1B—C1B—C21B	64.13 (19)
C2A—N1A—C1A—C21A	−74.36 (18)	C2B—N1B—C1B—C21B	−61.88 (19)
C6A—N1A—C2A—C3A	−63.95 (17)	C6B—N1B—C2B—C3B	66.43 (17)
C1A—N1A—C2A—C3A	173.49 (14)	C1B—N1B—C2B—C3B	−166.71 (14)
N1A—C2A—C3A—C7A	−151.02 (17)	N1B—C2B—C3B—C7B	148.23 (18)
N1A—C2A—C3A—C4A	28.7 (2)	N1B—C2B—C3B—C4B	−30.7 (2)
O1A—O1A—C4A—C5A	0.0 (3)	O1B—O1B—C4B—C5B	0.0 (2)
O1A—O1A—C4A—C3A	0.0 (3)	O1B—O1B—C4B—C3B	0.0 (3)
C7A—C3A—C4A—O1A	1.2 (3)	C7B—C3B—C4B—O1B	−2.5 (3)
C2A—C3A—C4A—O1A	−178.50 (16)	C2B—C3B—C4B—O1B	176.6 (2)
C7A—C3A—C4A—O1A	1.2 (3)	C7B—C3B—C4B—O1B	−2.5 (3)
C2A—C3A—C4A—O1A	−178.50 (16)	C2B—C3B—C4B—O1B	176.6 (2)
C7A—C3A—C4A—C5A	−177.55 (15)	C7B—C3B—C4B—C5B	175.28 (17)
C2A—C3A—C4A—C5A	2.7 (2)	C2B—C3B—C4B—C5B	−5.7 (3)
O1A—C4A—C5A—C14A	3.9 (3)	O1B—C4B—C5B—C14B	6.4 (3)
O1A—C4A—C5A—C14A	3.9 (3)	O1B—C4B—C5B—C14B	6.4 (3)
C3A—C4A—C5A—C14A	−177.34 (15)	C3B—C4B—C5B—C14B	−171.31 (17)
O1A—C4A—C5A—C6A	−179.84 (16)	O1B—C4B—C5B—C6B	−172.3 (2)
O1A—C4A—C5A—C6A	−179.84 (16)	O1B—C4B—C5B—C6B	−172.3 (2)
C3A—C4A—C5A—C6A	−1.1 (2)	C3B—C4B—C5B—C6B	9.9 (3)
C2A—N1A—C6A—C5A	65.66 (18)	C2B—N1B—C6B—C5B	−62.09 (18)
C1A—N1A—C6A—C5A	−171.47 (14)	C1B—N1B—C6B—C5B	171.20 (14)
C14A—C5A—C6A—N1A	143.83 (17)	C14B—C5B—C6B—N1B	−155.89 (17)
C4A—C5A—C6A—N1A	−32.1 (2)	C4B—C5B—C6B—N1B	22.7 (2)
C4A—C3A—C7A—C8A	174.85 (17)	C4B—C3B—C7B—C8B	−178.06 (18)
C2A—C3A—C7A—C8A	−5.5 (3)	C2B—C3B—C7B—C8B	3.0 (3)
C3A—C7A—C8A—C13A	−19.2 (3)	C3B—C7B—C8B—C13B	25.4 (3)
C3A—C7A—C8A—C9A	165.48 (18)	C3B—C7B—C8B—C9B	−157.34 (19)
C13A—C8A—C9A—C10A	0.3 (3)	C13B—C8B—C9B—C10B	−2.4 (3)
C7A—C8A—C9A—C10A	175.98 (16)	C7B—C8B—C9B—C10B	−179.87 (17)
C8A—C9A—C10A—C11A	0.2 (3)	C8B—C9B—C10B—C11B	1.5 (3)
C9A—C10A—C11A—C12A	0.1 (3)	C9B—C10B—C11B—C12B	0.3 (3)
C9A—C10A—C11A—Cl1A	−178.20 (14)	C9B—C10B—C11B—Cl1B	178.39 (14)
C10A—C11A—C12A—C13A	−0.9 (3)	C10B—C11B—C12B—C13B	−1.0 (3)
Cl1A—C11A—C12A—C13A	177.40 (14)	Cl1B—C11B—C12B—C13B	−179.10 (14)
C11A—C12A—C13A—C8A	1.4 (3)	C11B—C12B—C13B—C8B	0.0 (3)
C9A—C8A—C13A—C12A	−1.1 (3)	C9B—C8B—C13B—C12B	1.7 (3)
C7A—C8A—C13A—C12A	−176.41 (17)	C7B—C8B—C13B—C12B	178.97 (17)
C4A—C5A—C14A—C15A	178.45 (16)	C4B—C5B—C14B—C15B	178.23 (18)
C6A—C5A—C14A—C15A	2.5 (3)	C6B—C5B—C14B—C15B	−3.1 (3)
C5A—C14A—C15A—C16A	−153.04 (19)	C5B—C14B—C15B—C16B	160.85 (19)
C5A—C14A—C15A—C20A	28.7 (3)	C5B—C14B—C15B—C20B	−22.0 (3)
C20A—C15A—C16A—C17A	−1.7 (3)	C20B—C15B—C16B—C17B	2.8 (3)
C14A—C15A—C16A—C17A	179.90 (16)	C14B—C15B—C16B—C17B	−179.86 (16)
C15A—C16A—C17A—C18A	2.1 (3)	C15B—C16B—C17B—C18B	−1.8 (3)
C16A—C17A—C18A—C19A	−0.8 (3)	C16B—C17B—C18B—C19B	−0.8 (3)
C16A—C17A—C18A—Cl2A	178.13 (14)	C16B—C17B—C18B—Cl2B	178.72 (14)
C17A—C18A—C19A—C20A	−0.8 (3)	C17B—C18B—C19B—C20B	2.5 (3)
Cl2A—C18A—C19A—C20A	−179.76 (13)	Cl2B—C18B—C19B—C20B	−177.08 (14)
C18A—C19A—C20A—C15A	1.2 (3)	C18B—C19B—C20B—C15B	−1.5 (3)
C16A—C15A—C20A—C19A	0.0 (3)	C16B—C15B—C20B—C19B	−1.1 (3)
C14A—C15A—C20A—C19A	178.24 (16)	C14B—C15B—C20B—C19B	−178.24 (17)
N1A—C1A—C21A—C22A	120.53 (17)	N1B—C1B—C21B—C22B	85.3 (2)
N1A—C1A—C21A—C26A	−61.1 (2)	N1B—C1B—C21B—C26B	−94.30 (19)
C26A—C21A—C22A—C23A	−1.1 (3)	C26B—C21B—C22B—C23B	1.1 (3)
C1A—C21A—C22A—C23A	177.27 (16)	C1B—C21B—C22B—C23B	−178.52 (16)
C21A—C22A—C23A—C24A	0.7 (3)	C21B—C22B—C23B—C24B	−1.0 (3)
C22A—C23A—C24A—C25A	0.3 (3)	C22B—C23B—C24B—C25B	0.5 (3)
C23A—C24A—C25A—C26A	−0.9 (3)	C23B—C24B—C25B—C26B	−0.1 (3)
C24A—C25A—C26A—C21A	0.5 (3)	C24B—C25B—C26B—C21B	0.2 (3)
C22A—C21A—C26A—C25A	0.5 (2)	C22B—C21B—C26B—C25B	−0.7 (3)
C1A—C21A—C26A—C25A	−177.93 (16)	C1B—C21B—C26B—C25B	178.92 (17)

### Hydrogen-bond geometry (Å, °)

**Table:**

*D*—H···*A*	*D*—H	H···*A*	*D*···*A*	*D*—H···*A*
C16B—H16B···O1A	0.95	2.42	3.334 (2)	160
C14A—H14A···O1B	0.95	2.52	3.309 (2)	141
C16A—H16A···O1B	0.95	2.48	3.099 (2)	122
